# Trypsinization-dependent cell labeling with fluorescent nanoparticles

**DOI:** 10.1186/1556-276X-9-568

**Published:** 2014-10-13

**Authors:** Tetiana Serdiuk, Sergei Alekseev, Vladimir Lysenko, Valeriy Skryshevsky, Alain Géloën

**Affiliations:** 1 University of Lyon, CarMeN Laboratory, INSA de Lyon, UMR INSERM 1060, Lyon, France; 2 Institute of High Technologies, Taras Shevchenko National University of Kyiv, 64, Volodymyrska St., 01601 Kyiv, Ukraine; 3 Chemistry Faculty, Taras Shevchenko National University of Kyiv, 64, Volodymyrska St., 01601 Kyiv, Ukraine; 4 University of Lyon, Nanotechnology Institute of Lyon (INL), UMR 5270, CNRS, INSA Lyon, Villeurbanne F-69621, France

**Keywords:** Fluorescent nanoparticles, Cell labeling, Trypsin

## Abstract

Trypsin is often used to detach adhered cell subculture from a substrate. However, the proteolytic activity of trypsin may harm cells by cleaving the cell membrane proteins. The present study shows that cellular uptake of fluorescent nanoparticles is remarkably increased within 24 h after trypsinization. These results highlight the trypsin-induced protein digestion, provoking leaky cell plasma membrane which leads to the strongly enhanced cellular uptake of the nanoparticles. To prevent this effect, one should expose cells to the nanoparticle (NP)-based fluorescent labels at least 48 h after trypsinization.

## Background

Since the intracellular uptake of inorganic nanoparticles (NPs) has been discovered, NPs became a widely used tool for long-term fluorescent imaging of living cells [1-3]. So far, it has been successfully shown that the intensity of the intracellular uptake efficiency and localization of NPs inside the cells are strongly dependent on a set of various factors related to (i) NPs: size [4,5], surface shape [5,6], charge [7,8], and composition of protein corona [9] as well as to (ii) cell physiology: mitosis phase [10] and cell proliferation [11]. However, numerous points remain unclear in the process of interaction between live cells and few-nanometer fluorescent NPs. Detailed investigations of this interaction and deeper understanding of mechanisms responsible for it are important tasks of modern nanobiotechnology.

Cell culture treatment procedure is well-known to contain specific steps which are obviously stressful for cell, such as, for example, trypsinization. Trypsin is a protease catalyzing the hydrolysis of peptide bonds. It is used to break peptide bonds of the cell membrane proteins, in particular proteins responsible for cell adhesion. Trypsinization is necessary to detach adhered cells from a substrate. In this paper, we show how trypsin treatment strongly increases the permeability of the cell plasma membrane to the environmental NPs. Indeed, the trypsinization step results in an abnormal high intracellular uptake of the NPs within 24 h after the treatment. This leads to cell saturation even at low NP concentration and abolishes the dose-response fluorescence curve of the labeled cells as a function of NP concentration. That observation must be compulsorily taken into account when the cells are exposed to NPs shortly after the treatment with trypsin.

## Methods

**Figure 1:**
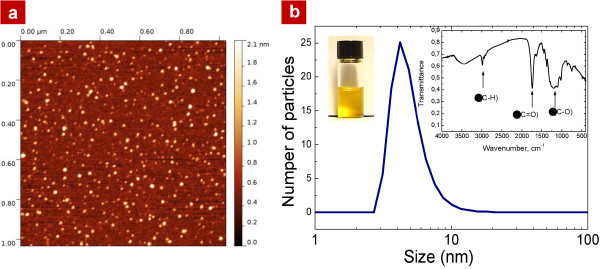
**AFM visualization and dynamic light scattering measurements of the obtained NPs. (a)** AFM image of the used CFO NPs; **(b)** size distribution of the CFO NPs, the left inset shows colloidal aqueous solution with the dispersed CFO NPs and the right inset gives an idea on their dominating chemical bonds.

Fluorescent carbon-based NPs were formed by means of electrochemical anodization of a low resistivity grade (<1 Ω cm) bulk 3C-SiC polycrystalline wafer. The etching process took place for 3 h at a current density of 25 mA/cm^2^ using a 1:1 HF (50%)/ethanol electrolyte. After the etching, a powder mixture mainly constituted by carbon fluoroxide (CFO) NPs and 3C-SiC porous nanostructures was formed. The nanopowder was then naturally dried, removed from the SiC wafer, mechanically grinded, and then dispersed in a Krebs buffer solution. The formed colloidal suspension was centrifuged at 10,000 × *g* for 5 min in order to collect only its top part containing very small (<10 nm) and homogeneously dispersed NPs which can be visualized on an AFM picture as shown in Figure 1a. Size distribution of the obtained NPs was estimated from dynamic light scattering measurements (see Figure 1b) and the average NPs size is found to be in the range 4 to 6 nm. FTIR spectrum shown as right inset in Figure 1b gives an idea about dominating chemical bonds (C-H, C = O, and C-O) taking place in the fabricated NPs. In addition, an elemental composition of the nanoparticles can be described as C_100.0_H_104.1_F_19.5_O_51.0_ brutto formula. The main surface chemical groups of the CFO NPs are supposed to be carboxylic and ester functionalities. Photoluminescence spectra of the CFO NPs are found to be centered at 550 nm under excitation at 400 nm.

The colloidal suspension described above was added to cell cultures of 3T3-L1 murine fibroblasts (American Type Culture Collection, Manassas, VA, USA), HSC-2 (a human oral squamous carcinoma line), and S-G (a human immortalized gingival epithelioid cell line, both kindly provided by Dr. Babich, Yeshiva University, New York). The cells were grown in Dulbecco’s modified Eagle’s medium supplemented with 10% newborn calf serum, 4 mM glutamine, 4 nM insulin (Actrapid Human; Novo Nordisk, Bagsvaerd, Denmark), 10 mM HEPES, 25 μg sodium ascorbate, 100 IU penicillin, 100 μg streptomycin, and 0.25 μg amphotericin B per milliliter, at 37°C in a water-saturated atmosphere with 5% CO_2_ in air, in a Heraeus incubator (BB16, Thermo Scientific, Villebon sur Yvette, France). The cells were incubated for 3 h in the presence of the CFO NPs (with concentrations ranging from 0.1 to 0.8 g/L) dispersed in Krebs bicarbonate buffer (pH =7.4). After the incubation period (3 h) and before their observation, the cells were rinsed twice in Krebs buffer to eliminate the NPs from their external environment. The fluorescence of the control and labeled cell cultures was observed by means of a fluorescence microscope (Leica DMI 4000B, Leica Microsystems, Wetzlar, Germany) with the following filter combination: UV/violet excitation (2.92 to 3.5 eV) with an observation spectral range <2.64 eV; acquisition time was 1 s; and magnification × 100.

For each image, luminosity per one cell was calculated by summing the intensity (in a range from 0 to 255) of each pixel on a fluorescence photo and dividing this result by the number of the fluorescent cells visible in the picture. The MathWorks MATLAB R2009b and Simulink software (MathWorks, Natick, MA, USA) were used for the image analysis.

## Results and discussion

**Figure 2:**
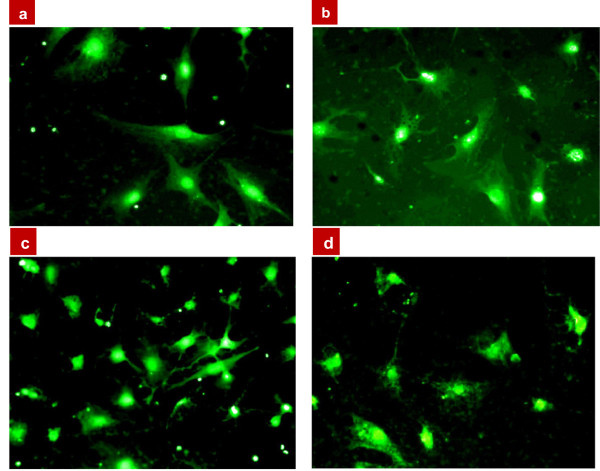
**Fluorescence microscope images of 3T3-L1 fibroblast cells labeled by the CFO NPs.** Cells were exposed to different concentrations at *t*_1_ = *t*_0_ + 24 h, where *t*_0_ corresponds to the end of trypsin treatment: **(a)** 0.1 mg/mL of CFO NPs, **(b)** 0.2 mg/mL, **(c)** 0.4 mg/mL, and **(d)** 0.8 mg/mL.

**Figure 3:**
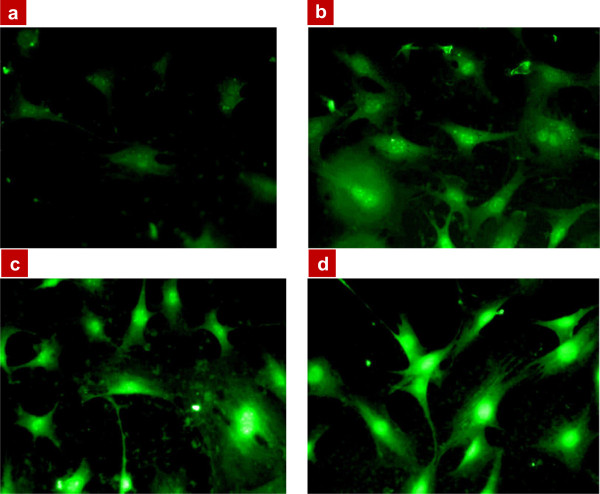
**Fluorescence microscope images of 3T3-L1 fibroblast cells labeled by the CFO NPs.** Cells were exposed to different concentrations at *t*_1_ = *t*_0_ + 48 h, where *t*_0_ corresponds to the end of trypsin treatment: **(a)** 0.1 mg/mL of CFO NPs, **(b)** 0.2 mg/mL, **(c)** 0.4 mg/mL, and **(d)** 0.8 mg/mL.

**Figure 4:**
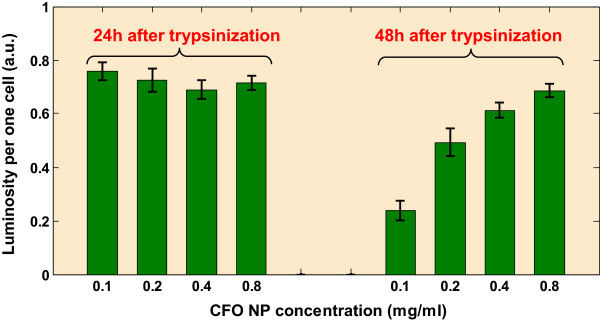
**Quantitative summary of observed effects illustrated by previous images.** Luminosity per one fibroblast cell as function of the NP concentrations for *t*_1_ = *t*_0_ + 24 h and *t*_2_ = *t*_0_ + 48 h.

**Figure 5:**
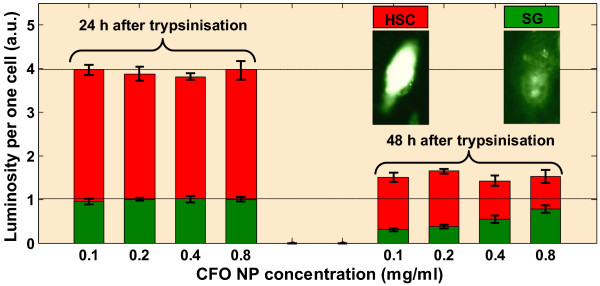
**Luminosity per one cell as a function of the NP concentrations.** NPs have been added for *t*_1_ = *t*_0_ + 24 h and *t*_2_ = *t*_0_ + 48 h: green bars - epithelial healthy cells (SG), red bars - epithelial cancer cells (HSC).

CFO NPs have been chosen for our experiments due to a set of their major advantages, such as few-nanometer size, relatively narrow size distribution, sufficiently intense green luminescence under UV excitation, high intracellular uptake without additional chemical functionalization, and natural targeting of cell nuclei. In previous studies [7,11,12], we have always observed, under the same experimental conditions of the present study, an intranuclear localization of NP SiC. Numerous arguments show that the localization of NP SiC is intranuclear. During cell division, DNA is replicated and the nucleus divides. It would be extremely difficult to argue that NPs remain extracellular and follow the changes of the nucleus while remaining fixed on the cell membrane. We also performed confocal microscopy and showed that NP SiC has an intranuclear localization.Figure 2 shows fluorescence images of the 3T3-L1 cells exposed to different concentrations of the CFO NPs, 24 h after the trypsin treatment. One can state complete independence of the cell fluorescence intensity on the NP concentration. Indeed, the fluorescence level is already high at the lowest used NP concentration (0.1 mg/mL).Figure 3 shows the general view of the same cells exposed to increasing concentrations of the CFO NPs, 48 h after the trypsin treatment. A clear dose-response effect can be observed: the higher NP concentration gives the stronger fluorescence of the labeled cells.Figure 4 quantitatively summarizes the observed effects qualitatively illustrated by the previous fluorescence images.The same experiment has also been carried out on two human cell lines: (i) cancer cell line (HSC) and (ii) its healthy equivalent (SG). Figure 5 shows the luminosity per one labeled cell for the case of the human epithelial cells (SG, green bars) and oral squamous carcinoma cell line (HSC, red bars) when the NPs have been added 24 or 48 h after the trypsin treatment, at the same concentrations range used for the 3T3-L1 cells. The insets show typical fluorescence images of the labeled cells for both tested human cell lines: healthy and cancer one.

The most important observation can be done in the case when the SG cells are exposed to the CFO NPs within 24 h following the trypsinization: no characteristic dose-response dependence is observable. The fluorescence intensity starts to increase with the increasing NP concentration only when cells are exposed at least 48 h after the trypsinization procedure.

As one can see, in the case of the HSC cancer cells, no dose-response dependence is observable either 24 or 48 h after the trypsinization step. The absence of any dose-response effect results from the fact that the cancer cells proliferate rapidly and also because the NP uptake is significantly increased in the proliferating cells [11]. In addition, a significant luminescent difference between the labeled cancer cells treated 24 and 48 h after trypsinization takes place. Indeed, the NP uptake efficiency is much higher when the cancer cells are exposed to the CFO NPs within 24 h than after 48 h. Here again, this observation confirms that trypsin leads to increase of the cell membrane permeability to the NPs.

All these results indicate on the fact that trypsin renders the plasma membranes porous and highly permeable to the used NPs. Consequently, 48 h is necessary to restore the normal permeability of the plasma membrane. This observation is of a crucial importance because most of the studies reported in literature and devoted to penetration of fluorescent NPs into living cells are carried out on proliferating cells, suggesting that they have been trypsinized for a short time before the NP exposure. Our observation correlates well to the fact that trypsin is known to alter the cell plasma membrane. Indeed, cell transient increase during the rounding up, possibly due to the detachment of the ‘feet’ holding the cells onto the substrate, has been reported [13]. Experimental data support a leak formation by SH group modification of skeletal proteins [14].

Studies of the effects, such as NP uptake, cell survival, cell migration, etc., can be strongly misleading if cells are trypsinized shortly before their exposure to NPs. At least 48 h is necessary for the cells to recover normal plasma membrane functions. From another side, trypsin can be used to facilitate the entry of NPs inside the cells. Indeed, the transient increase in cell permeability in response to the trypsin exposure may be a valuable method for introducing large molecules or NPs.

## Conclusions

In summary, the experimental results presented above show for the first time that trypsinization, which is widely used in the process of cell culture treatment, favors the penetration of NPs inside the cells when they are exposed less than 24 h after the trypsin treatment. A simple way to prevent such an important effect is to wait at least 48 h after trypsinization procedure before cell exposure to NPs.

## Competing interests

The authors declare that they have no competing interests.

## Authors' contributions

TS performed cell growth, nanoparticle fabrication, fluorescence microscopy measurements, and data analysis. SA carried out structural and chemical composition studies of the CFO nanoparticles. VL performed general data analysis and contributed to the discussion of the obtained data. VS contributed to the discussion of the obtained data. AG also performed the cell growth, ensured general coordination of the project, and contributed to the data analysis. All authors participated in writing the manuscript and approved its final version.
